# Common mental disorders and HIV status in the context of DREAMS among adolescent girls and young women in rural KwaZulu-Natal, South Africa

**DOI:** 10.1186/s12889-021-10527-z

**Published:** 2021-03-10

**Authors:** Nondumiso Mthiyane, Guy Harling, Natsayi Chimbindi, Kathy Baisley, Janet Seeley, Jaco Dreyer, Thembelihle Zuma, Isolde Birdthistle, Sian Floyd, Nuala McGrath, Frank Tanser, Maryam Shahmanesh, Lorraine Sherr

**Affiliations:** 1 Africa Health Research Institute, Durban, KwaZulu-Natal South Africa; 2 Institute for Global Health, University College London, London, UK; 3 MRC/Wits Rural Public Health & Health Transitions Research Unit (Agincourt), University of the Witwatersrand, Johannesburg, South Africa; 4 Department of Epidemiology & Harvard Center for Population and Development Studies, Harvard T.H. Chan School of Public Health, Boston, MA USA; 5 University of KwaZulu-Natal, Durban, South Africa; 6 London School of Hygiene and Tropical Medicine, London, UK; 7 University of Southampton, Southampton, UK; 8 University of Lincoln, Lincoln, UK

**Keywords:** HIV prevention, Adolescents, Women, Mental health, South Africa

## Abstract

**Background:**

HIV affects many adolescent girls and young women (AGYW) in South Africa. Given the bi-directional HIV and mental health relationship, mental health services may help prevent and treat HIV in this population. We therefore examined the association between common mental disorders (CMD) and HIV-related behaviours and service utilisation, in the context of implementation of the combination DREAMS (Determined, Resilient, Empowered, AIDS-free, Mentored and Safe) HIV prevention programme in rural uMkhanyakude district, KwaZulu-Natal. DREAMS involved delivering a package of multiple interventions in a single area to address multiple sources of HIV risk for AGYW.

**Methods:**

We analysed baseline data from an age-stratified, representative cohort of 13–22 year-old AGYW. We measured DREAMS uptake as a count of the number of individual-level or community-based interventions each participant received in the last 12 months. CMD was measured using the validated Shona Symptom Questionnaire, with a cut off score ≥ 9 indicating probable CMD. HIV status was ascertained through home-based serotesting. We used logistic regression to estimate the association between CMD and HIV status adjusting for socio-demographics and behaviours.

**Results:**

Probable CMD prevalence among the 2184 respondents was 22.2%, increasing steadily from 10.1% among 13 year-old girls to 33.1% among 22 year-old women. AGYW were more likely to report probable CMD if they tested positive for HIV (odds ratio vs. test negative: 1.88, 95% confidence interval: 1.40–2.53). After adjusting for socio-demographics and behaviours, there was evidence that probable CMD was more prevalent among respondents who reported using multiple healthcare-related DREAMS interventions.

**Conclusion:**

We found high prevalence of probable CMD among AGYW in rural South Africa, but it was only associated with HIV serostatus when not controlling for HIV acquisition risk factors. Our findings highlight that improving mental health service access for AGYW at high risk for HIV acquisition might protect them. Interventions already reaching AGYW with CMD, such as DREAMS, can be used to deliver mental health services to reduce both CMD and HIV risks. There is a need to integrate mental health education into existing HIV prevention programmes in school and communities.

**Supplementary Information:**

The online version contains supplementary material available at 10.1186/s12889-021-10527-z.

## Background

Despite the success of antiretroviral treatment, HIV still affects many South Africans [1]. Adolescent girls and young women (AGYW) face disproportionately high HIV risks, including of acquisition [2]. The drivers of HIV acquisition among AGYW relate to a complex array of biological, psychological, interpersonal and structural factors, leading to what has been termed a unique vulnerability [3]. A recent Kenyan study showed that AGYW who were older, out of school and not living with their parents were more likely to engage in risky sexual behaviours and less likely to undertake HIV testing [4]. In South Africa, a 2012 nationally representative population-based survey highlighted the need to address multiple concerns – including economic, educational, alcohol-related and sexual factors – in order to mitigate young females’ vulnerability to HIV [5].

Mental health problems can both be a cause and a result of hardships and challenges, such as those relating to HIV [6]. There is a growing awareness of the broader HIV epidemic’s enormous mental health burden [7–9]. However, few studies have examined the role of mental health in the context of the complex drivers of HIV-related risk [10, 11]. People living with HIV (PLHIV) show elevated rates of depression, anxiety and trauma [12–14], which may be due to HIV-related stigma [15, 16], the direct disease burden, or the impact of an HIV diagnosis on quality of life and relationships. People with mental health challenges are also at greater risk of HIV exposure and acquisition [17–19]. A recent survey of adults in Zimbabwe found that psychological distress was associated with increased sexual risk behaviour among seronegative individuals, and with reduced adherence to treatment for PLHIV [17]. In sub-Saharan Africa (SSA), the situation is made more complex by the relative lack of resources for mental health support [20] – despite emerging data on effective interventions [21] – and the need for locally validated, culturally relevant screening tools to identify mental health problems.

This limited understanding of the interplay of HIV and mental health in SSA is even more acute in the adolescent population. At best, mental health needs are usually only considered within the context of providing psychosocial support [22]. In a recent WHO scoping exercise, mental health was seen as a priority for adolescents in low- and middle-income countries [23], but there remains a need to establish the mental health vulnerabilities of PLHIV, those affected by the disease and most-at-risk adolescents [24]. Mental health considerations are crucial in understanding both pathways to risk [25] and how emotional support can help ameliorate mental health burden in adolescents [26]. All these findings point to a need to measure and provide support for mental health in the HIV response.

One important recent HIV intervention for AGYW in SSA has been the DREAMS (Determined, Resilient, Empowered, AIDS-free, Mentored and Safe) Partnership. DREAMS was created in 2014 by the United States President’s Emergency Plan for AIDS Relief (PEPFAR) and aimed to substantially impact the HIV epidemic in AGYW in SSA [27]. DREAMS is a multi-component HIV prevention intervention focused on reducing HIV incidence among AGYW living in high HIV prevalence and incidence settings. It includes interventions aimed at biological, behavioural, social and structural sources of HIV acquisition risk [28]. The integration of mental health considerations into DREAMS might offer an opportunity to substantially expand mental health service provision to AGYW in SSA.

In South Africa, high levels of youth unemployment, poverty and violence are likely to cause mental health issues in young people; these issues will worsen their vulnerability to HIV and may have a negative impact on HIV prevention intervention implementation. Few studies have explored mental health-related HIV risk behaviour, or prevalence of common mental disorders (CMD) among PLHIV [29]; the literature is skewed towards high income settings, adults, and key populations such as men who have sex with men. Furthermore, mental health measures are not often culturally adapted. There is no study that has looked at CMD among young people in the context of intensive HIV prevention interventions. We hypothesise that mental health among AGYW is associated with HIV serostatus and related risk factors, and may affect the ability of adolescents to take up interventions that improve resilience to HIV. We therefore examined the prevalence of CMD, and their association with HIV-related risk and DREAMS service utilization, in a cohort of South African AGYW.

## Methods

### Study design

We used baseline data from a cohort of AGYW in rural KwaZulu-Natal, South Africa. This cohort was formed as part of the impact evaluation of the DREAMS Partnership as rolled out in uMkhanyakude district [30]. UMkhanyakude is one of the poorest districts in South Africa and has among the highest HIV prevalence and incidence rates in the country [31]. DREAMS was implemented in uMkhanyakude by local partners and community-based organizations who work closely with government departments to deliver defined intervention packages [32]. The DREAMS impact evaluation sought to measure: (1) population-level changes over time in HIV incidence and socio-economic, behavioural and health outcomes among AGYW and young men (before, during, after DREAMS); and (2) causal pathways linking uptake of DREAMS interventions to ‘mediators’ of change, such as empowerment (including mental health), to behavioural and health outcomes [30]. The DREAMS evaluation was conducted in the southern section of Africa Health Research Institute’s (AHRI) Population Intervention Platform surveillance area, which covers ~800km^2^ with a population of approximately 140,000 members of 12,000 households [33]. The area is largely rural with one town of population ~ 30,000.

For the DREAMS impact evaluation, a closed cohort of 3013 AGYW aged between 13 and 22 at baseline was selected using a random stratified sampled from the AHRI census of age-eligible household residents in 2017 [30]. The sample was stratified by age (13–17; 18–22) and by 45 geographic areas. Baseline interviews were conducted between May 2017 and February 2018 in isiZulu using a structured quantitative questionnaire programmed in REDCap [34]. The interview included questions on socio-demographics, general health, exposure to DREAMS interventions, sexual relationships and violence. For sexual behaviour questions, participants were given a tablet computer to complete a self-interview; the fieldworker was available to provide support as needed.

### Measures

#### Outcome

Our primary outcome was ‘probable Common Mental Disorder’ (CMD), measured by the 14-item Shona Symptom Questionnaire (SSQ-14) [35, 36], which has been validated in a high HIV prevalence setting in Zimbabwe [37], and used in SSA as a robust and culturally relevant mental health measure [38]. The SSQ-14 asks whether participants have experienced a range of symptoms ever in the past seven days, including sleep disturbance, lack of concentration, irritability, slowness in activity, stomach ache, lack of energy, hopelessness and thoughts of suicide. A total score is obtained by summing affirmative answers. We calculated a binary variable using the validated cut-off for probable CMD in Zimbabwe of ≥9 [35], and assessed the scale’s internal reliability using Cronbach’s Alpha.

#### Exposures

We considered a wide range of variables previously shown to predict CMD and other mental health outcomes in young people in SSA. We included several socio-demographic variables: current occupation (in school, employed, neither); household urbanicity; household relative wealth (tertiles of the first component of a principal component analysis based on household asset ownership and access to safe drinking water and sanitation); personal cellphone ownership; food insecurity (any report of reducing the size of food potions or skipping meals by any member of a household because there was not enough money to buy food in the past 12 months); and migration status (ever having moved home since the age of 13). We also included sexual and substance use variables: alcohol use (never drank, ever drank in lifetime, drank within the last month); any history of gravidity; self-reported knowledge of HIV status (yes or no); and HIV serostatus based on dried blood spot samples provided for anonymous testing by participants. We additionally used a 15-item checklist about lifetime experience of physical, psychological and sexual violence perpetrated by men (details in Supplementary Table 1) to generate a binary variable of any reported experience of gender-based violence (GBV).

For DREAMS, we grouped interventions offered locally into two categories [30]. First, seven individual-level, healthcare-related interventions: HIV testing and counselling; family planning services; adolescent and youth friendly health services; condom promotion and provision; sexually transmitted infection screening; emergency contraception; and post-violence care. Second, nine family or community-level interventions: AGYW safe spaces; mentoring programmes; training programmes for social asset building, business and vocational skills, and financial literacy; cash transfers to AGYW and families; parenting programmes; and school-based HIV education. For each category we generated a count of the number of interventions each respondent reported receiving in the last 12 months, collapsing three and above into one level.

### Statistical analysis

All participants with complete SSQ-14 data were included in analysis. After describing variables using proportions and 95% confidence intervals (CI), we used logistic regression to conduct first bivariate, then age-adjusted and finally multivariable analysis for each exposure variable and probable CMD. We tested for multicollinearity and possible interactions between independent variables before fitting a multivariable logistic regression model. All analyses were performed using Stata version 15 (StataCorp LP, College Station, Texas USA).

### Ethics

The DREAMS Partnership impact evaluation protocol was approved by the University of KwaZulu-Natal Biomedical Research Ethics Committee (BFC339/19), the London School of Hygiene & Tropical Medicine Research Ethics Committee (REF11835) and the AHRI Somkhele Community Advisory Board. For participants aged below 18 years, written parental consent and participant assent was required; participants aged 18 years or older provided a written consent.

## Results

**Fig. 1:**
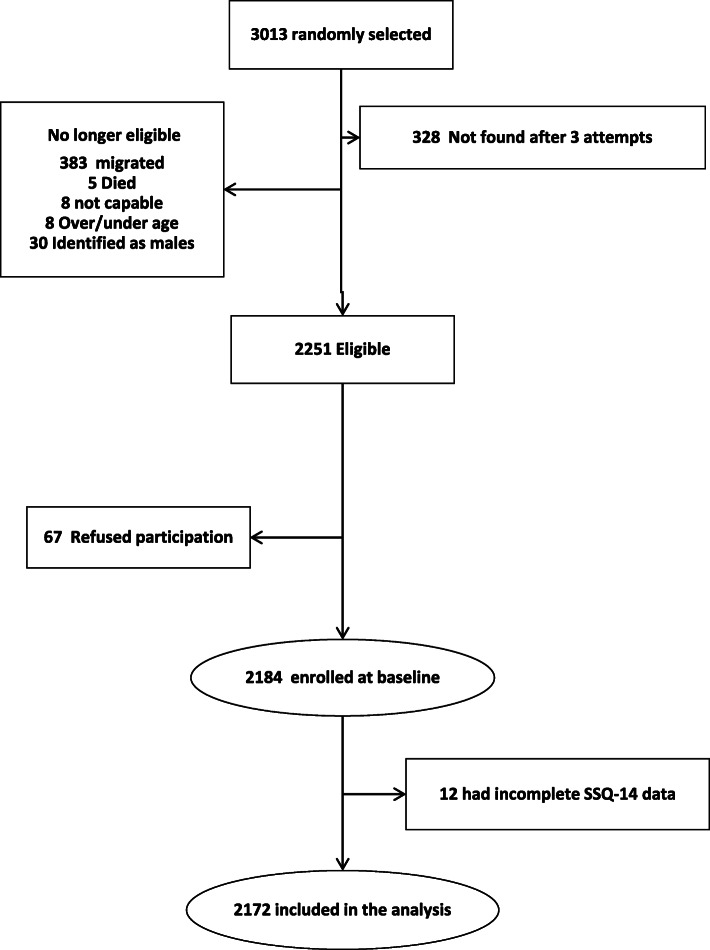
Participant flowchart

Of 3013 eligible sampled individuals, 2251 (74.7%) were located, of whom 67 declined to participate. Of the 2184 respondents, 2172 (99.4%) had complete SSQ-14 data (Fig. 1). A majority of AGYW resided rurally and were currently still in school; 18.5% had moved home since age 13 (Table 1). About one-quarter had ever drunk alcohol, and 9.7% had done so in the last month; one-third reported food insecurity in the last 12 months. More than one-third had ever experienced either psychological, physical or sexual gender-based violence. Almost half of respondents had tested for HIV and knew their status; 10.7% were seropositive. Uptake of one or two DREAMS interventions was similar for community-level and individual-level interventions, but more respondents (23.0%) had used three or more community-level interventions.

**Table 1:** Characteristics of adolescent girls and young women by Common Mental Disorder status

	n	% of all	% with probable CMD
**Age group**			
13–14	459	21.1	11.8
15–17	685	31.5	19.0
18–19	473	21.8	25.8
20–22	555	25.6	31.9
**Occupation**			
Neither in school nor employed	509	23.5	28.7
In school	1633	75.3	20.1
Employed	28	1.3	25.0
**Socio-economic status**			
Lowest third	723	35.1	21.3
Middle third	741	35.9	21.6
Highest third	598	29.0	23.1
**Urbanicity**			
Rural	1382	64.2	20.0
Peri-urban/urban	771	35.8	26.2
**Food insecurities**			
No	1494	68.8	18.7
Yes	678	31.2	29.9
**Has cellphone**			
No	795	36.6	15.6
Yes	1376	63.4	26.1
**Migrated ever since age 13**			
Never	1771	81.5	20.6
Within PIPSA	201	9.3	26.9
External migration	200	9.2	32.5
**Ever been or currently pregnant**			
No	1570	74.0	18.3
Yes	553	26.0	31.8
**Knows own HIV status**			
No	1192	55.1	18.2
Yes	970	44.9	27.3
**HIV status**			
Seronegative	1789	82.7	21.1
Seropositive	233	10.7	33.5
Unknown	150	6.9	18.7
**Ever experienced gender-based violence**			
No	1404	64.6	18.4
Yes	768	35.4	29.3
**Ever drunk alcohol**			
Never	1645	75.9	19.3
Ever but not last month	310	14.3	28.1
Drank last month	211	9.7	37.0
**Community-level DREAMS interventions used**			
None	643	29.6	25.0
One	652	30.0	22.9
Two	378	17.4	21.4
Three or more	499	23.0	18.4
**Individual-level DREAMS interventions used**			
None	756	34.8	15.5
One	767	35.3	22.8
Two	401	18.5	26.4
Three or more	248	11.4	34.3

*CMD* Common mental disorders. Total sample is 2172. *P*-values for *χ*^2^ tests of equality of proportion with CMD across all categories of a variable

**Fig. 2:**
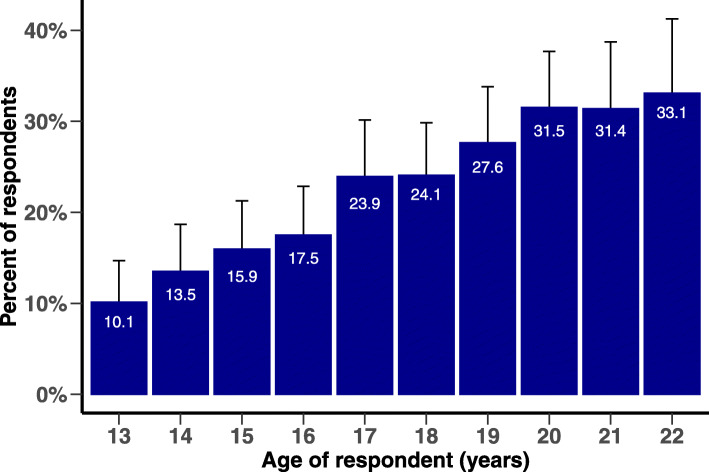
Prevalence and 95% confidence interval of Common Mental Disorder by age

**Fig. 3:**
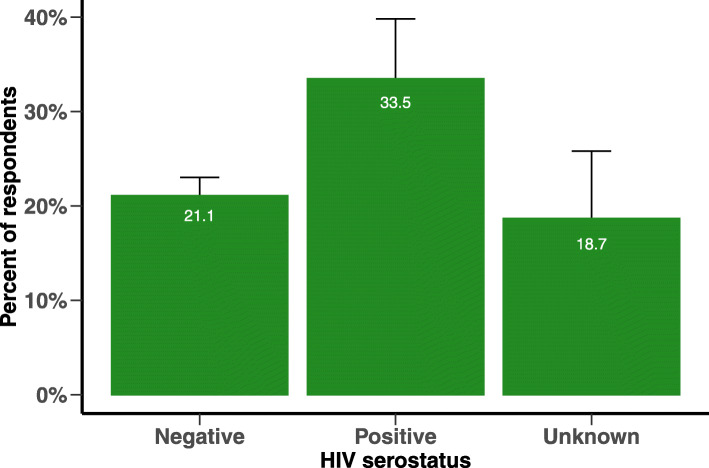
Prevalence and 95% confidence interval of Common Mental Disorder by HIV serostatus

A total of 483 respondents (22.2%) had SSQ-14 scores meeting the probable CMD criterion. Probable CMD prevalence increased linearly with age from 10.1% at age 13 to 33.1% at age 22 (Fig. 2) and was significantly more common among AGYW who tested positive for HIV (33.5%) (Fig. 3). AGYW with probable CMD differed significantly from others on several characteristics (Table 2, column 1). Respondents living in peri-urban or urban areas, who were not in school, who reported a history of food insecurity and had ever migrated outside of Population Intervention Platform surveillance area were more likely to report probable CMD. Those who have ever been pregnant, who knew their HIV status and tested positive for HIV, who reported having experienced any GBV and have ever drunk alcohol were also more likely to have probable CMD. Respondents who reported receiving more individual-level, and fewer community-level, DREAMS interventions in the past 12 months were more likely to have probable CMD. Several of these associations (notably school attendance, migration, pregnancy, HIV status knowledge and serostatus, and receipt of individual-level DREAMS interventions) reflected partial or substantial confounding by age (Table 2, column 2).

**Table 2:** Predictors of Common Mental Disorders in adolescent girls and young women

	Bivariate		Age adjusted		Multivariate	
	OR	95% CI	OR	95% CI	OR	95% CI
**Age group**						
13–14	1				1	
15–17	1.76	1.25–2.47			1.52	1.03–2.23
18–19	2.61	1.84–3.70			1.93	1.23–3.03
20–22	3.51	2.51–4.91			2.41	1.46–3.98
**Occupation**						
In school	0.63	0.50–0.79	1.13	0.85–1.50	1.21	0.83–1.76
Employed	0.83	0.34–1.99	0.78	0.32–1.87	0.56	0.18–1.75
**Socio-economic status**						
Lowest third	1		1		1	
Middle third	1.05	0.82–1.35	1.04	0.81–1.35	1.05	0.80–1.37
Highest third	1.11	0.85–1.44	1.17	0.89–1.52	1.23	0.91–1.65
**Urbanicity**						
Rural	1		1		1	
Peri-urban/urban	1.42	1.15–1.74	1.44	1.17–1.78	1.32	1.04–1.67
**Food insecurities**						
Yes	1.85	1.50–2.28	1.58	1.28–1.97	1.72	1.35–2.19
**Has cellphone**						
Yes	1.91	1.52–2.40	1.26	0.97–1.64	1.15	0.86–1.53
**Migrated ever since age 13**						
Within PIPSA	1.42	1.02–1.98	1.07	0.76–1.51	0.98	0.66–1.46
External migration	1.86	1.35–2.56	1.26	0.90–1.76	1.22	0.83–1.79
**Ever been or currently pregnant**						
Yes	2.08	1.67–2.59	1.39	1.07–1.81	1.29	0.94–1.76
**Knows own HIV status**						
Yes	1.69	1.38–2.07	1.28	1.03–1.59	1.06	0.82–1.37
**HIV status**						
Seronegative	1		1		1	
Seropositive	1.88	1.40–2.53	1.47	1.08–1.99	1.26	0.89–1.77
Unknown	0.86	0.56–1.32	0.72	0.46–1.10	0.66	0.41–1.07
**Ever experienced gender-based violence**						
Yes	1.84	1.50–2.26	1.97	1.59–2.43	1.84	1.46–2.32
**Ever drunk alcohol**						
Ever but not last month	1.63	1.24–2.15	1.56	1.18–2.07	1.48	1.08–2.01
Drank last month	2.46	1.81–3.33	2.27	1.67–3.10	2.18	1.54–3.06
**Community-level DREAMS interventions used**						
One	0.89	0.69–1.14	1.28	0.97–1.68	1.09	0.77–1.54
Two	0.82	0.60–1.11	1.27	0.91–1.76	1.14	0.76–1.71
Three or more	0.68	0.51–0.90	1.13	0.82–1.56	1.00	0.67–1.50
**Individual-level DREAMS interventions used**						
One	1.61	1.25–2.09	1.28	0.97–1.68	1.19	0.87–1.62
Two	1.96	1.46–2.64	1.34	0.98–1.85	1.10	0.74–1.61
Three or more	2.85	2.05–3.95	1.96	1.38–2.78	1.69	1.10–2.61

*OR* Odds ratio; *CI* Confidence interval. Where not shown, reference category is ‘no’, ‘none’ or ‘never’

In multivariable analysis, probable CMD was significantly more common among AGYW living in non-rural areas and those with food insecurity, and among those who had experienced GBV (adjusted odds ratio [aOR]: 1.84, 95% confidence interval [CI]: 1.46–2.32) or who reported drinking alcohol either in the last month (aOR: 2.18, 95%CI: 1.54–3.06) or ever (aOR: 1,48, 95%CI: 1.08–2.01). AGYW who tested positive for HIV remained more likely to have probable CMD than those who tested negative, but this association was attenuated (aOR: 1.26, 95%CI: 0.89–1.77). Probable CMD remained more common among those who had received three or more individual-level DREAMS interventions (aOR: 1.69, 95%CI: 1.10–2.61).

## Discussion

We found high levels of probable CMD in AGYW in this rural South African setting, exceeding 30% by age 20. CMD prevalence increased significantly with age and was higher among individuals living with HIV. We also found that CMD was strongly associated with HIV-related risk factors, including violence, alcohol, food insecurity, migration and living in an urban vs rural area. AGYW who used at least three DREAMS individual-level interventions were significantly more likely to have CMD, suggesting that DREAMS programmes are reaching vulnerable AGYW and are thus a potential platform to screen for and deliver mental health interventions.

Similar to previous studies [39, 40], older AGYW were more likely to have probable CMD compared to younger AGYW. This finding suggests that appropriate interventions to prevent mental disorders in adolescents should be made available at an early age to reduce the burden of untreated and comorbid mental disorders. The high levels of CMD in the older group (above 18 years) may also be driven by limited employment opportunities and migration to a new place for work. Out-of-school AGYW in South Africa are not age-eligible for government non-contributory grants once above 18 years, at the same time that familial material support is typically withdrawn. Given very high youth unemployment, this generates substantial financial strain. All these challenges that young people face as they transition out of school, often without a strong peer support, are likely to increase CMD risk.

Our findings are consistent with previous evidence that CMD are more common among those living with HIV, especially those newly diagnosed with HIV [17, 41–44]. Previous studies among adult PLHIV in KwaZulu-Natal reported high levels of self-stigma or stigmatisation by healthcare workers, which increase their risk of CMD [15, 16]. Stigma can also lead to non-adherence to HIV treatment and cause worse outcomes if not diagnosed early. CMD may also be a reaction to some of the drivers of HIV infection, such as poverty, alcohol use and isolation. The complex mosaic of risk and mental health requires exploration in greater depth. The cross-sectional nature of this data does not allow us to determine the course of CMD; future longitudinal data will allow us to explore whether elevated CMD precedes HIV infection, is a reaction to diagnosis, or both.

Our finding that probable CMD is higher in urban areas accords with a previous national study of adolescents [45]. This association may reflect urban living being stressful, or that migrated from rural areas has led to diminished support alongside increased economic and social pressures. We also found that food insecurity, which is linked to poverty, was associated with increased odds of probable CMD, in line with evidence from previous studies [46, 47], including evidence from Zimbabwe that people with suicidal ideation who reported a history of food insecurity were more likely to have CMD [48].

Probable CMD was highly prevalent among AGYW who had ever been pregnant, but this association weakened after accounting for age suggesting that the concurrent transition to both adulthood and parenthood for young girls is associated with higher risk of developing mental disorders [49]. In South Africa, most adolescent pregnancies are unplanned, which can lead to CMD due to lack of support from family, partner neglect or social exclusion by their peers [50, 51]. Screening of pregnant young women for CMD during their ante-natal care visits can help identify young women with mental health needs.

Probable CMD was associated with the use of multiple individual-level DREAMS interventions, suggesting that AGYW with mental health problems have greater health care needs. DREAMS interventions in uMkhanyakude were delivered by multiple implementing partners with varying reach and quality [32]. As this was not a trial, participants who received multiple healthcare services may have higher needs (including elevated mental health needs) than those who received fewer services. It is therefore hard to determine the causality of this finding.

There is a need for better integration of mental health services into primary and community-based care in this setting. Successful models of lay provider delivery of such care in Zimbabwe may be worth considering. For example, the friendship bench programme, which uses lay health workers, has shown to be effective in addressing mental health burden and supplementing the treatment gap in CMD [48, 52]. A recent systematic review in low-and middle-income countries settings provides clear guidance of evidence-based interventions such as group support psychotherapy and peer-support counselling which could be integrated into community and primary care services interventions [21].

Exposure to GBV was associated with probable CMD, as seen in previous studies in South Africa and elsewhere [53–55]. We also found that current use of alcohol was strongly associated with CMD; again consistent with previous studies [56–58], and with CMD changing behaviour in young adolescence, resulting in increased alcohol and other substance use at a later age. Social interventions are needed to protect young women against GBV and improve social resilience during transitions – in part to prevent young people resorting to drugs and alcohol as a coping mechanism. Alternatively, drugs and alcohol may be part of experimentation and peer influence in this age group and those with CMD may be particularly vulnerable to influence and escalating use. High levels of CMD found in this setting may prevent young people from accessing health care, leading to poor health outcomes. For example, previous studies found mental ill-health is associated with poor adherence to ART among PLHIV [59], and therefore more likely to affect other health outcomes.

Our study had some limitations. First, our data were cross-sectional, and thus we were not able to determine the temporal ordering of health and socio-demographic conditions; follow-up work with this cohort and elsewhere may help disentangle such pathways. Second, much of our data was self-reported and thus may be subject to social desirability and recall bias, potentially leading to underreporting of stigmatized conditions including CMD or over-reporting of food insecurity, for example. In this context, the substantial prevalence of CMD should be considered conservative. Third, SSQ-14 has not been specifically validated for use in adolescents younger than 18 years, therefore, the CMD prevalence in adolescents maybe underestimated. A cut-off of 5 or more has been found to be more effective than the original cut-off of 8 or more in capturing depression cases in adolescents [36]. Finally, it is difficult to be sure how widely our results can be generalized. We would expect our findings to be applicable for other settings in sub-Saharan Africa where HIV and CMD are common, although associations may differ in poorer, or more urban, areas than uMkhanyakude. Analysis of cohorts elsewhere in South Africa and beyond will be needed to confirm the generalizability of our findings. Despite the limitations, our study has some strengths. We used a large representative sample which allowed us to explore the difference in CMD prevalence between age groups. This study also adds important data on CMD prevalence and its risk factors in young people using SSQ-14 that was validated in adults in a similar setting.

## Conclusions

Common mental disorders are prevalent among adolescent girls and young women in this rural South African setting, and are associated with poverty, HIV and violence. Multilevel HIV prevention interventions reached AGYW with probable CMD, highlighting that community-based programmes provide an opportunity for CMD prevention, screening and treatment. The high levels of CMD we observed in young people may lead to escalating risk and negative short- and long-term impacts on well-being if left unmanaged. Programmes and provisions that include non-stigmatizing and youth-friendly screening, referral and resources for mental health should be considered for AGYW. Integrating mental health education into HIV prevention programmes in schools and communities would help raise awareness about mental health issues and may identify affected young people at an early stage.

## Supplementary Information

**Additional file 1: Supplementary Table 1.** Description of types of violence experience by Common Mental Disorder status.

## Data Availability

The dataset supporting the conclusions of this article is available in the AHRI Data Repository (10.23664/ahri.dreams.nested.cohort.baseline) [60].

## Abbreviations

AGYW: Adolescent Girls and Young Women
CMD: Common Mental Disorders
PEPFAR: President’s Emergency Plan for AIDS Relief
DREAMS: Determined, Resilient, Empowered, AIDS-free, Mentored and Safe
SSA: Sub-Saharan Africa
PLHIV: People Living with HIV
GBV: Gender-Based Violence
