# Supplementary material for: Common mental disorders and HIV status in the context of DREAMS among adolescent girls and young women in rural KwaZulu-Natal, South Africa
Source: BMC Public Health. 2021 Mar 10;21:478. doi: 10.1186/s12889-021-10527-z (PMC7945212; doi:10.1186/s12889-021-10527-z)
Supplement: Supplementary file 1 — Additional file 1: Supplementary Table 1. Description of types of violence experience by Common Mental Disorder status. [file 12889_2021_10527_MOESM1_ESM.docx]

Supplementary Table 1. Description of types of violence experience by Common Mental Disorder status

|  | **Overall** | |  | **AGYW with probable CMD** | |  |
| --- | --- | --- | --- | --- | --- | --- |
|  | **N** | **%** |  | **n** | **%** | **p-value** |
| **Any psychological violence** |  |  |  |  |  |  |
| **No** | **1705** | **78.5** |  | **331** | **19.4** |  |
| **Yes** | **467** | **21.5** |  | **152** | **32.5** | **<0.001** |
| Say or do something that humiliate you |  |  |  |  |  |  |
| No | 1958 | 90.1 |  | 403 | 20.6 |  |
| Yes | 214 | 9.9 |  | 80 | 37.4 | <0.001 |
| Threaten to hurt/harm you or someone close to you. |  |  |  |  |  |  |
| No | 2028 | 93.4 |  | 424 | 20.9 |  |
| Yes | 144 | 6.6 |  | 59 | 41 | <0.001 |
| Insult you or make you feel bad about yourself |  |  |  |  |  |  |
| No | 1874 | 86.3 |  | 395 | 21.1 |  |
| Yes | 298 | 13.7 |  | 88 | 29.5 | 0.001 |
|  |  |  |  |  |  |  |
| **Any physical violence** |  |  |  |  |  |  |
| **No** | **1589** | **73.2** |  | **312** | **19.6** |  |
| **Yes** | **583** | **26.8** |  | **171** | **29.3** | **<0.001** |
| Push, shake you or throw something at you |  |  |  |  |  |  |
| No | 1964 | 90.4 |  | 428 | 21.8 |  |
| Yes | 208 | 9.6 |  | 55 | 26.4 | 0.125 |
| Slap you |  |  |  |  |  |  |
| No | 1791 | 82.5 |  | 353 | 19.7 |  |
| Yes | 381 | 17.5 |  | 130 | 34.1 | <0.001 |
| Twist your arm or pull your hair |  |  |  |  |  |  |
| No | 1981 | 91.2 |  | 420 | 21.2 |  |
| Yes | 191 | 8.8 |  | 63 | 33 | <0.001 |
| Punch you with his fist or something that could hurt you |  |  |  |  |  |  |
| No | 2075 | 95.5 |  | 445 | 21.4 |  |
| Yes | 97 | 4.5 |  | 38 | 39.2 | <0.001 |
| Kick, drag or hit you |  |  |  |  |  |  |
| No | 2045 | 94.2 |  | 440 | 21.5 |  |
| Yes | 127 | 5.8 |  | 43 | 33.9 | 0.001 |
| Tried to strangle you or burn you |  |  |  |  |  |  |
| No | 2121 | 97.7 |  | 463 | 21.8 |  |
| Yes | 51 | 2.3 |  | 20 | 39.2 | 0.003 |
| Threatened to stab you with knife or other weapon |  |  |  |  |  |  |
| No | 2088 | 96.1 |  | 449 | 21.5 |  |
| Yes | 84 | 3.9 |  | 34 | 40.5 | <0.001 |
| Attack you with a weapon |  |  |  |  |  |  |
| No | 2136 | 98.3 |  | 467 | 21.9 |  |
| Yes | 36 | 1.7 |  | 16 | 44.4 | 0.001 |
|  |  |  |  |  |  |  |
|  |  |  |  |  |  |  |
|  |  |  |  |  |  |  |
|  |  |  |  |  |  |  |
| **Any sexual violence** |  |  |  |  |  |  |
| **No** | **1975** | **90.9** |  | **418** | **21.2** |  |
| **Yes** | **197** | **9.1** |  | **65** | **33** | **<0.001** |
| Touched you in a sexual way |  |  |  |  |  |  |
| No | 2061 | 94.9 |  | 444 | 21.5 |  |
| Yes | 111 | 5.1 |  | 39 | 35.1 | 0.001 |
| Tried to force you to have sex with him but didn't succeed |  |  |  |  |  |  |
| No | 2076 | 95.6 |  | 450 | 21.7 |  |
| Yes | 96 | 4.4 |  | 33 | 34.4 | 0.003 |
| Physically force you to have sex with him |  |  |  |  |  |  |
| No | 2130 | 98.1 |  | 465 | 21.8 |  |
| Yes | 42 | 1.9 |  | 18 | 42.9 | 0.001 |
| Force you to perform sexual activities with him |  |  |  |  |  |  |
| No | 2124 | 97.8 |  | 462 | 21.8 |  |
| Yes | 48 | 2.2 |  | 21 | 43.8 | <0.001 |
